# On the Origin of Neutrophil Extracellular Traps in COVID-19

**DOI:** 10.3389/fimmu.2022.821007

**Published:** 2022-03-11

**Authors:** Michal Pastorek, Martin Dúbrava, Peter Celec

**Affiliations:** ^1^ Institute of Molecular Biomedicine, Faculty of Medicine, Comenius University, Bratislava, Slovakia; ^2^ Department of Geriatric Medicine, Faculty of Medicine, Comenius University, Bratislava, Slovakia; ^3^ Institute of Pathophysiology, Faculty of Medicine, Comenius University, Bratislava, Slovakia; ^4^ Department of Molecular Biology, Faculty of Natural Sciences, Comenius University, Bratislava, Slovakia

**Keywords:** COVID – 19, thrombosis, neutrophil, extracellular traps (ETs), DAMPs

## Abstract

Despite ongoing vaccination COVID-19 is a global healthcare problem because of the lack of an effective targeted therapy. In severe COVID-19 manifesting as acute respiratory distress syndrome, uncontrolled innate immune system activation results in cytokine deregulation, damage-associated molecular patterns release upon tissue damage and high occurrence of thrombotic events. These pathomechanisms are linked to neutrophil function and dysfunction, particularly increased formation of neutrophil extracellular traps (NETs). While the association of NETs and severity of COVID-19 has been shown and proved, the causes of NETs formation are unclear. The aim of this review is to summarize potential inducers of NETs formation in severe COVID-19 and to discuss potential treatment options targeting NETs formation of removal.

## Introduction

SARS-CoV-2 causes much more than just COVID-19. The world is still facing huge socio-economic problems that will likely persist much longer than the pandemic itself (1). Experts agree that a population-wide vaccination is the most effective weapon in the fight against SARS-CoV-2, but its application is not trivial in today’s world (2, 3). Due to the current state of misinformation, those who would not be vaccinated represent a significant portion of the population in many countries, although the situation is dynamic and changes with the number of vaccines that have been approved (2, 4–6). Unfortunately, if not enough people are vaccinated, the pandemic will not stop (7). If such a scenario occurs, the only remaining solution will be targeted and effective treatment of patients with severe COVID-19 (8). Several treatment strategies have already been proposed, but most of them do not decrease COVID-19 mortality, but at best reduce the time of hospitalization (9–11), with some even being ineffective and harmful (12). So far, the most successful approach seems to be immunosupressive therapy (11, 13), but to design the best treatment is only possible if pathogenesis of the disease is known in detail (14). And there are still gaps to fill.

## Thrombosis in COVID-19

At the beginning of the pandemics, COVID-19 was almost exclusively viewed in the context of lung damage, and therefore artificial lung ventilation appeared to be a key therapeutic intervention (15). However, initial results from China, Italy, and the United States showed, that mortality of COVID-19 patients admitted to ICU that were in the need of mechanical ventilation was greater than 90% (16–18). Although the data were not so alarming in other countries later (17, 19), it was clear that the pathophysiology of COVID-19 required a more comprehensive view. A partial explanation was provided by a study published in the Lancet, where the authors showed that patients infected with SARS-CoV-2 show endothelial dysfunction due to endothelial inflammation, so-called endothelitis (20). The damaged endothelium facilitates coagulation and thrombus formation, whether in large vessels or in small arterioles and capillaries (21). This thrombosis and subsequent coagulopathy cannot, of course, be resolved by artificial lung ventilation and additional oxygenation (22). Thrombotic complications were indeed found to be one of the major issues in treating critically ill ICU patients with COVID-19 (23). It has become clear, that identifying the initiators and drivers of thrombosis is vital.

## Neutrophil Extracellular Traps

DNA is found inside the nucleus and mitochondria of the cell and as the primary information-carrying molecule is protected by several membranes from external potentially damage-causing factors (24). The same membranes, however, protect the DNA also from release outside of the cell. Nevertheless, various types of cell death might lead to DNA release into the extracellular space (25). During inflammation, a significant source of this cell-free DNA (cfDNA) comes from a specific type of neutrophil response - the so-called NETosis, a process that results in the formation of neutrophil extracellular traps (NETs) (26). NETs are web-like structures composed of DNA-histone complexes decorated by antimicrobial proteins and enzymes such as myeloperoxidase (MPO), neutrophil elastase (NE), cathelicidin, calprotectin and many others (27). In fact, their composition varies and has been reported to be dependent on the stimulus that activates neutrophils and initiates NETs release (28, 29).

## Induction of NETs Formation

Formation of NETs was initially discovered as a response of neutrophils to the presence of bacteria and immediately, their role in prevention of pathogen dissemination was recognized (26). Since then, the list of bacteria that can induce the formation of NETs has substantially grown (30–37). Neutrophils are also capable of sensing the size of the stimulus and can selectively form NETs in response to larger pathogens such as fungi and parasites (38–44). Interestingly, NETs formation was also found to be stimulated by viruses (Hantavirus, hRSV, HIV, influenza) but their role in antiviral defense *in vivo* remains unresolved (45–52). While NETs might potentially restrain virus particles and their individual components possess antiviral properties, NETs were not found to be induced during mild influenza infection and mice that are incapable of their formation do not display increased susceptibility to influenza virus (51, 52). On the other hand, NETs most likely mediate pathology of severe viral infections, where virus-induced tissue damage allows subsequent bacterial overgrowth that together with endogenous stimuli drives NETs release (53, 54). Pathogens are recognized by neutrophils through a variety of pattern recognition receptors (PRR’s) such as toll-like receptors (TLR’s) 2, 4, 7, 8 and 9, dectins 1 and 2 and can also induce NETs formation *via* activation of calcium signaling by calcium ionophores (55).

Sterile stimuli are also capable of NETs induction and even NETs themselves have been described to induce more NETs (56, 57). If excessive NETs formation damages endothelium or other tissue, neutrophils detect parts of free mitochondria that are released from dead cells as damage-associated molecular patterns (DAMPs) (58). More than 10 years ago, Carl J Hauser and colleagues found that despite billions of years of evolution, the immune system still recognizes mitochondria as bacteria (59). This may be important in the crush syndrome, in polytrauma, where patients end up in a septic shock-like condition even though they do not have any confirmed microbial infection (60). Individual mitochondrial DAMPs activate different receptors. Mitochondrial DNA contains unmethylated CpG islets that are ligands for the Toll-like receptor 9 (TLR9) (61–63). Formylated peptides and proteins of mitochondrial origin are recognized by formyl peptide receptors (FPR1-2) (64, 65) and saturated cardiolipin is able to activate TLR4 mediated signaling (66, 67). During viral pneumonia induced breakdown of pneumocytes, endothelocytes, pulmonary megakaryocytes or during the formation of NETs by neutrophils, free mitochondria are released (68, 69). These can subsequently activate the immune system either as intact organelles or as their individual mitochondrial DAMPs. Similar mechanism might be at play in severe COVID-19 infection.

Another endogenous stimulus such as activated platelets can induce NETs through the interaction of High mobility group box 1 (HMGB1) with the receptor for advanced glycation end products (RAGE) or TLR4 and P-selectin through binding to P-selectin glycoprotein ligand (70–72). NETs formation is also induced by the binding of anti-nuclear or anti-neutrophil antibodies and immobilized immune complexes to FcγRIIIb receptor (73–76), and even nanoparticles, cholesterol and monosodium urate crystals can stimulate NETs formation (77–82). Finally, phorbol 12-myristate 13-acetate (PMA) triggers NETs formation independently of any receptor *via* activation of protein kinase C (PKC) and production of reactive oxygen species (ROS) and is often used as positive control for NETs induction (30). All of the pathogenic, as well as non-infectious stimuli capable of NETs induction are listed in the **Table 1**.

**Table 1:** Known pathogenic as well as sterile NETs inducers, corresponding receptors they interact with, along with a pathway the are independent of regarding NETs formation.

	stimulus	receptor	signaling independent of	reference
**pathogenic**	bacteria	FPR1, FPR2, TLR4, TLR9	–	(26, 30–37)
	fungi	Dectin 1, 2	not known	(38–42)
	viruses	TLR7, TLR8, ACE2	not known	(45–52, 83, 84)
	parasites	TLR2, TLR4	NOX2	(43, 44)
	ionophores	none	ERK, NOX2	(36, 37)
**sterile**	platelets	RAGE, PSGL1, TLR2, TLR4	NOX2	(70–72)
	mitochondria	TLR4, TLR9, FPR1, FPR2	–	(58, 59, 63–67)
	immune complexes	FcγRIIIb	NOX2	(33, 73–76)
	crystals and nanoparticles	none	PAD4	(77–81)
	PMA	none	PAD4	(30)

Formation of NETs is a double-edged sword (85). While being an extremely potent part of the antimicrobial defense, the emerging NETs must also be rapidly removed. Otherwise, the NETs activate other neutrophils and immunocompetent cells contributing to the inflammation that generates more NETs (55, 86). This creates a vicious cycle that is a key component in the pathogenesis of diseases as diverse as preeclampsia, sepsis or rheumatoid arthritis (71, 87, 88), and data suggests, that it is important for COVID-19 as well.

## NETs Drive Thrombosis in COVID-19

The hypothesis that neutrophils and NETs are implicated in the formation of thrombi during severe SARS-CoV-2 infection has been proposed several times (89–94). Blood myeloperoxidase-DNA complex levels (i.e. NETs) were identified as a biomarker of an early response to SARS-CoV-2 infection, suggesting that circulating NETs are involved in COVID-19 pathology (95). Since then, several studies found that the production of NETs is increased in COVID-19 and their concentration is associated with severity of the disease and thrombosis (96–100), and NETs were found to be predominantly located in the lower respiratory tract of critically-ill patients (101). Skendros and his colleagues even proposed a mechanism of NETs induced thrombosis in COVID-19, where SARS-CoV-2 triggered complement activation leads to thrombin induced expression of tissue factor (TF) in neutrophils, which results in TF rich pro-coagulatory NETs (100). Increased NETs formation during SARS-CoV-2 infection has also been linked to ischemic stroke, underlying the importance of therapy focused on the inhibition of NETs formation (102). The fact that several studies and meta-analyses identified neutrophilia as one of the predictors of COVID-19 severity and an increased neutrophil to lymphocyte ratio has high predictive value if present at the beginning of the infection further underscores the role of neutrophils in early stages of COVID-19 pathology (103–107). In addition, the dysregulation of myeloid populations resulting in immature or dysfunctional neutrophils was found to be characteristic for developing severe, but not mild COVID-19 (108, 109). Lastly, genetic predisposition might also affect NETs mediated COVID-19 pathology. Genome-wide association study investigating genetic variants associated with circulating NETs levels in plasma revealed a variant in *TMPRSS13* gene coding a type II transmembrane serine protease to be significantly associated with increased level of MPO-DNA complexes (110). Interestingly, the same protease TMPRSS13 was reported to enhance cellular uptake and replication of SARS-CoV-2, making it an interesting target for future investigation (111). Other study identified a variant on 3p21.31 region associated with increased respiratory failure risk in COVID-19 that enhances expression of leucine zipper transcription factor like 1 gene (*LZTFL1*). *LZTFL1* regulates a viral response pathway and is associated with epithelial-mesenchymal transition and it is possible that this epithelial dysfunction is driven by neutrophil extracellular traps (112, 113).

## Mechanisms of NETs Induction in COVID-19

Soon after it was found that SARS-CoV-2 infection results in the formation of neutrophil extracellular traps, the search for possible mechanisms of NETs induction in COVID-19 has begun. Arcanjo and his colleagues were the first to describe that both live, and heat-inactivated SARS-CoV-2 virus cultivated on and isolated from Vero cells could induce NETs formation at surprisingly low concentrations (83). Possible mechanism of SARS-CoV-2 induced NETs formation was later proposed by Veras and his colleagues. They reported that live, but not formaldehyde inactivated SARS-CoV-2 virus induces the formation of NETs and their induction is dependent on virus binding to neutrophil angiotensin converting enzyme (ACE2) receptor, again at interestingly low multiplicity of infection rate of 1 (84). Additionally, neutrophil elastase – a NETs component, is able to cleave S protein, resulting in an easier SARS-CoV-2 entry into the cell through ACE2, potentially increasing virus infectivity and its ability to stimulate immune response (114). Thus, as was already proposed, NETs formation might be induced by SARS-CoV-2 virus and at the same time increase its infectivity, making NETs and neutrophil elastase promising treatment targets (115). Whether these findings apply to a situation *in vivo* remains to be elucidated.

**Figure 1:**
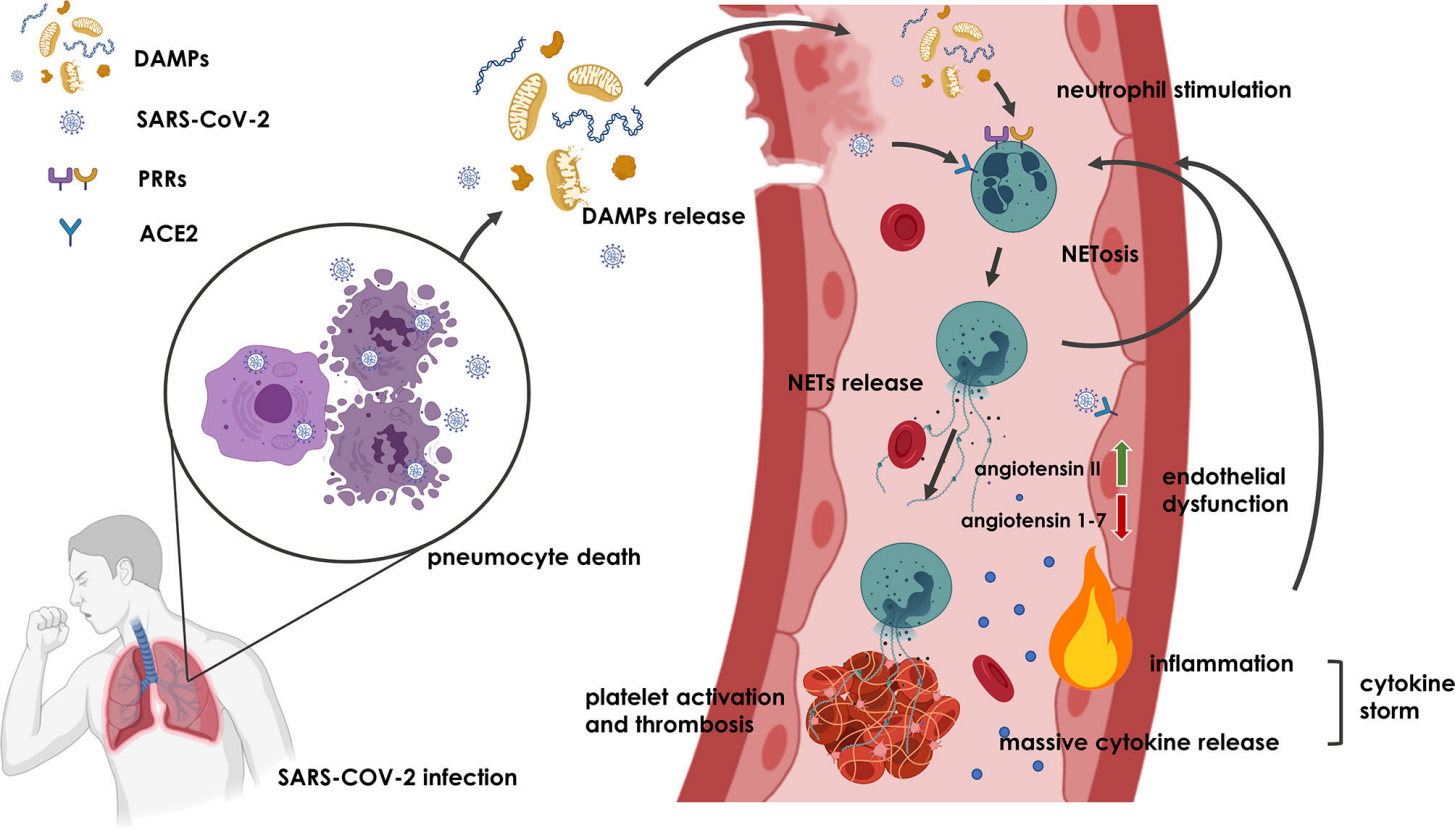
Potential mechanism underlying NETs formation and thrombosis induction in COVID-19. Upon SARS-CoV-2 infection, pneumocyte death and endothelial dysfunction result in the release of DAMPs and SARS-CoV-2 into extracellular space, where they bind to PRRs and ACE2 receptors and initiate activation of neutrophils and formation of NETs. NETs that are not removed from the circulation induce more NETs in a vicious circle and cause thrombosis and inflammation that might even lead to cytokine storm. Additionally, binding of SARS-CoV-2 on ACE2 receptor of endothelial cells may promote angiotensin II and angiotensin 1-7 imbalance leading to endothelial dysfunction and inflammation, which further contributes to NETs induction and thrombus formation. Figure was created with BioRender.com.

One possible factor linking endothelial dysfunction and deregulation of NETs formation with COVID-19 might be angiotensin 1-7, a product of ACE2, which functions as a key receptor for SARS-CoV-2 (116). Binding of the virus to this receptor leads to a reduction in the production of angiotensin 1-7 as a ligand of the Mas receptor (117, 118). The resulting imbalance between increasing angiotensin II and decreasing angiotensin 1-7 can stimulate endothelial dysfunction, an inflammatory response, induce NETs and thrombus formation (119–121). The consequences of these pathomechanisms are consistent with the histopathology of COVID-19 (122). Compared to influenza, lung necropsies in patients with COVID-19 showed similar diffuse alveolar damage but much more pronounced thrombosis with microangiopathy. Microthrombi were up to 9 times more frequent in the pulmonary circulation of COVID-19 when compared to influenza (122). Proposed mechanisms of NETs formation and induction of thrombosis in COVID-19 are illustrated in **Figure 1**.

## Targeting NETs Formation

Whether a neutrophil decides to form a NET depends on the context, i.e. also on the size, number and structural properties of the potential inducers (123). Understanding NETosis on a molecular level is extremely important, as the knowledge of signaling pathways involved in NETs induction will enable for selective inhibition of NETs formation, rather than just unspecific attenuation of inflammation. As was mentioned above, both pathogenic and sterile stimuli activate neutrophils through binding of various membrane and intracellular receptors and *via* MEK–extracellular-signal-regulated kinase (ERK) and protein kinase C (PKC) induce the production of ROS. ROS then activate MPO, which triggers oxidative activation of NE required for the degradation of actin cytoskeleton and subsequent histone processing upon NE nuclear translocation (41, 124, 125). Histone citrullination by protein-arginine deiminase type 4 (PAD4) further enhances chromatin decondensation and after mixing with cytoplasmatic components and permeabilization of the plasma membrane, NET is released into the extracellular space (30, 126–128).

To date, several compounds that target components of this pathway have been suggested as a potential intervention in COVID-19, most notable of them being Chloramidine, an inhibitor of PAD4 and NE inhibitor Sivelestat (ONO-5046), that has already been approved for the treatment of ARDS in Japan (92). While Sivelestat improves pulmonary function and oxygen saturation in ARDS patients, meta-analysis of completed clinical trials did not show improvement in survival of patients with ARDS (129). Currently, new generation of NE inhibitors (Lonodelestat, Alvelestat, CHF6333 and Elafin) have entered clinical trials, albeit neither NE nor PAD4 inhibitors are currently tested in clinical trials investigating COVID-19. Other, less specific drugs that could inhibit neutrophil recruitment or indirectly attenuate NETs formation such as Colchicine, Disulfiram, Anakinra, N-Acetyl Cysteine, Azithromycin, Aspirin, Cyclosporine A and Metformin are being clinically evaluated in COVID-19 but only two will inspect the effect of intervention on NETs formation (92). One retrospective study will examine the effect of Anakinra and the other examined the effect of disulfiram, but no results are currently available (NCT04594356, NCT04594343). Finally, hydroxychloroquine that interferes with NETs formation through inhibition of TLR9 has been proposed as a therapeutical intervention for COVID-19, although it has already been shown that it does not improve clinical outcome and mortality of patients with COVID-19 (130, 131).

Recently, mtDNA has been identified as an activator of cyclic GMP-AMP synthase (cGAS)-Stimulator of interferon genes (STING) signaling that drives aberrant type I interferon (type I IFN) response in COVID-19 (132). Moreover, pharmacological inhibition of STING improved disease outcome in a murine model of SARS-CoV-2 induced lung inflammation. Since type I IFN is also known to be an inducer of NETs formation, therapeutical targeting of DAMPs that are released from dead pneumocytes after SARS-CoV-2 infection should also be considered (133, 134). In fact, it has already been proposed, that cell-free mitochondria constitute a potential treatment target, since inhibition of their recognition by neutrophils could result in decreased neutrophil reactivity and NETs formation (135).

Another possible therapeutic strategy is to focus on the removal of NETs. NETs clearance is important for preventing sterile inflammation and thrombosis and is carried out by monocytes and macrophages, but also depends on the plasma nuclease activity (136). Because of histones, antimicrobial peptides and other proteins that bind DNA with high affinity, NETs may be partially resistant to deoxyribonuclease (137, 138). Additionally, anti-NET antibodies found in the plasma of COVID-19 patients likely also stabilize NETs and impair their clearance (139). Nevertheless, exogenous administration of recombinant deoxyribonuclease 1 has already been shown to decrease the concentration of plasma levels of cell free DNA and NETs *in vitro* and may be used as a potential therapeutic intervention (140). There are currently 8 registered clinical trials evaluating NETs in COVID-19 patients (NCT04409925, NCT04541979, NCT05139901, NCT04359654, NCT04402970, NCT04817332, NCT04594356, NCT04594343). Of those, NCT04594356, NCT04594343 were mentioned above and will investigate the effect of Anakinra and Disulfiram, and NCT04817332 evaluates the effect of protease inhibitor Brensocatib, that is expected to reduce NE activity. The remaining five are investigating the effect of recombinant human DNase 1 (rhDNase 1) on NET quantity, with NCT04402970 having already published results (141). In this study, treatment with rhDNase 1 was associated with decreased DNA-MPO complexes (i.e. NETs) in lungs as well as improved oxygenation. This study was however limited by its small sample size of 30 patients, and while a small decrease in mortality was observed upon rhDNase 1 treatment, it was not statistically significant and a more extensive trial would be warranted. All of the currently available as well as proposed treatments targeting NETs are listed in the **Table 2**.

**Table 2:** Compounds that degrade or inhibit the formation of NETs and their corresponding targets with proposed mechanism of action in relation to clinical trials with COVID-19 patients.

tested in	compound	target	mode of action	clinical trial identifier
**clinical trials evaluating NETs**	rhDNase 1	DNA	DNA degradation	NCT04409925, NCT04402970, NCT04541979, NCT05139901, NCT04359654
	Anakinra	IL-1β	IL-1 receptor antagonist	NCT04817332
	Disulfiram	Gasdermin A	Gasdermin A inhibition	NCT04594343
	Brensocatib	NE	inhibition of NE activity	NCT04817332
**clinical trials not evaluating NETs**	Azithromycine	Cytokines	inhibition of neutrophil migration	–
	Hydroxychloroquine	TLR9	increase of lysosomal pH	–
	Colchicine	Tubulin	disruption of microtubule assembly	–
	Aspirin	Cyclooxygenase 1 and 2	inhibition of platelet aggregation	–
	Metformin	mTORC1 and AMPK	AMPK activator	–
	N-acetyl cysteine	ROS	antioxidant attenuating ROS mediated signaling	–
	Cyclosporine A	Cytophilin	calcineurine pathway inhibitor	–
**not a subject of COVID-19 clinical trials**	Chloramidine	PAD4	inhibition of PAD4 activity	N/A
	Sivelestat, Lonodelestat, Alvelestat, CHF6333, Elafin	NE	inhibition of NE activity	N/A

NA, not applicable.

Increased concentration of NETs components and cfDNA were negatively associated with clinical outcomes, indicating that NETs formation should be potentially evaluated not only as a novel target for therapeutic interventions, but could also be used as a clinical biomarker (142). A case study by Zuo and colleagues found remnants of NETs such as cfDNA, citrullinated histone H3, myeloperoxidase and its complexes in patient sera were associated with higher risk of thrombosis, in spite of previous prophylactic anticoagulation (143). While this phenomenon should be further explored, these results suggest that standard anticoagulation treatment may not be sufficient and targeting NETs formation and promoting their degradation should be prioritized.

Today, it still remains unclear what induces the formation of NETs during SARS-CoV-2 infection, why geriatric and not immunosuppressed patients are at higher risk of death from COVID-19, and how to best intervene to avoid the negative consequences of increased NETs production. Since the early outbreak in Wuhan, old age was found to be a major risk factor for mortality of COVID-19 patients (144). While it has been hypothesized, that increased risk of thrombotic complications is attributed to individuals with specific genetic conditions that favor the release of NETs and are therefore predisposed for abnormal coagulation (145), so far, no studies have stratified COVID-19 patients ex ante based on NETs formation. Whether elderly people and those with underlying health problems such as diabetes or asthma are at the highest risk of developing severe COVID-19 because of altered neutrophil function and NETs formation remains to be determined.

## Conclusion

NETs research is in an exciting phase. While the evidence for the procoagulatory properties of NETs and their involvement in the COVID-19 pathology is growing stronger, insight into the mechanisms initiating their formation is still lacking. To develop targeted therapies focused on NETs inhibition is only possible if the factors that are involved in their induction are elucidated, and that requires extensive preclinical studies followed by clinical trials. This work presents current knowledge on the stimuli that might activate neutrophils and induce the formation of NETs during SARS-CoV-2 infection and highlights possible treatment options for COVID-19, but also for several other pathologies with shared pathogenesis involving NETs formation. Many unknowns need to be resolved, but understanding the complexities of NETs formation *in vivo* would be beneficial beyond the current pandemic.

## Author Contributions

MP, MD, and PC conceptualized and wrote the manuscript. All authors contributed to the article and approved the submitted version.

## Funding

The authors are supported by Slovak research and development agency (grant number PP-COVID-20-0016).

## Conflict of Interest

The authors declare that the research was conducted in the absence of any commercial or financial relationships that could be construed as a potential conflict of interest.

## Publisher’s Note

All claims expressed in this article are solely those of the authors and do not necessarily represent those of their affiliated organizations, or those of the publisher, the editors and the reviewers. Any product that may be evaluated in this article, or claim that may be made by its manufacturer, is not guaranteed or endorsed by the publisher.
